# Interferon gamma applied ex vivo restores function to neutrophils from critically ill patients

**DOI:** 10.1136/thorax-2025-223280

**Published:** 2025-08-28

**Authors:** Cameron J Lake, Jonathan Scott, Marie-Hélène Ruchaud-Sparagano, John H Thompson, Fiona Dewar, Polina Yarova, Wendy Funston, Richard CH Davidson, Kathryn M Musgrave, Stephen E Wright, Ian Clement, Alistair I Roy, Wezi Sendama, Jason Powell, Daniel Brooks, Chung Mun Alice Lin, Kristen Davies, Thomas P Hellyer, Anthony J Rostron, A John Simpson

**Affiliations:** 1 Translational and Clinical Research Institute, Newcastle University, Newcastle upon Tyne, UK; 2 Respiratory Medicine, Newcastle Upon Tyne Hospitals NHS Foundation Trust, Newcastle upon Tyne, UK; 3 Haematology, Newcastle Upon Tyne Hospitals NHS Foundation Trust, Newcastle upon Tyne, UK; 4 Anaesthesia and Critical Care, Newcastle Upon Tyne Hospitals NHS Foundation Trust, Newcastle upon Tyne, UK; 5 Integrated Critical Care Unit, Sunderland Royal Hospital, Sunderland, UK; 6 Department of Paediatric Otolaryngology, Great North Children’s Hospital, Newcastle Upon Tyne, UK

**Keywords:** Neutrophil Biology, Bacterial Infection, Critical Care

## Abstract

**Introduction:**

Critically ill patients commonly develop acquired neutrophil dysfunction, which increases susceptibility to intensive care unit-acquired infection (ICU-AI). This study aimed to assess whether interferon gamma (IFN-γ) can restore function in dysfunctional neutrophils from critically ill patients and to uncover potential underlying mechanisms.

**Methods:**

This was an observational cohort study. Neutrophils were isolated from whole blood donated by critically ill patients (n=31) in four separate teaching hospital intensive care units (ICUs). Neutrophils were subsequently treated with recombinant human IFN-γ or vehicle for 1 hour following either Fc gamma receptor (FcγR) blockade, selective inhibition of the gamma isoform of phosphoinositide 3-kinase (PI3K-γ) or vehicle control for 30 min. Neutrophil phagocytosis, bacterial killing, superoxide generation, phagocytic receptor expression and small Rho GTPase activity were assessed. Neutrophil dysfunction was defined as <50% of cells ingesting 2 or more zymosan particles in a phagocytosis assay.

**Results:**

IFN-γ significantly improved phagocytosis (control 36.5%, IFN-γ 56.0%), bacterial killing (control 31.6%, IFN-γ 82.1%) and superoxide generation (2.8-fold increase relative to control) in dysfunctional neutrophils. IFN-γ also increased the activity of the small GTPases, Rac and Cdc42 (2.4-fold and 1.5-fold increase relative to control, respectively) in dysfunctional neutrophils. Selective inhibition of PI3K-γ prevented the IFN-γ-mediated improvement of phagocytosis (IFN-γ 62.5%, with inhibitor 27.9%), bacterial killing (IFN-γ 82.1%, with inhibitor 30.5%) and superoxide generation (IFN-γ 2.8-fold change relative to control, 0.7 with inhibitor). The IFN-γ-mediated improvement of bacterial killing in dysfunctional neutrophils was also prevented by FcγR blockade (IFN-γ 82.1%, FcγR inhibition 28.7%).

**Conclusions:**

In critically ill patients with known acquired neutrophil dysfunction, ex vivo application of IFN-γ consistently improved a range of neutrophil effector functions.

**WHAT IS ALREADY KNOWN ON THIS TOPIC**

- Interferon gamma is used clinically to improve neutrophil function in people with chronic granulomatous disease, and in small case series interferon gamma has shown promising results in critically ill patients with sepsis. However, little is known about whether interferon gamma can improve the function of neutrophils from critically ill patients.

**WHAT THIS STUDY ADDS**

- Interferon gamma significantly improves key neutrophil effector functions associated with pathogen clearance.

**HOW THIS STUDY MIGHT AFFECT RESEARCH, PRACTICE OR POLICY**

- The ability of ex vivo interferon gamma to restore function to neutrophils from critically ill patients suggests it may be worth exploring the efficacy and safety of interferon gamma therapy in critically ill patients with demonstrable acquired neutrophil dysfunction.

## Introduction

 Among patients admitted to intensive care units (ICUs), the incidence of new, ICU-acquired infections (ICU-AIs) is estimated at 8%–22%.^1^,^5^ ICU-AI is associated with increased mortality and length of ICU stay.^1^ Neutrophils are key effector cells in the recognition, engulfment and destruction of bacterial and fungal pathogens. Approximately 30% of patients in ICUs acquire a functional impairment of neutrophil phagocytosis induced by their critical illness.^2 3^ The potential to restore neutrophil function pharmacologically raises the possibility that treatments could be developed to prevent ICU-AI in the most susceptible patients, without the requirement for excessive antibiotics.^6^

Interferon gamma (IFN-γ) is a pleiotropic type 2 interferon and immune cytokine that is licensed for clinical use in chronic granulomatous disease (CGD), in which it significantly improves neutrophil function and reduces intercurrent infection.^7^,^9^ CGD is caused by dysfunction of neutrophil NADPH oxidase, but IFN-γ has been shown to have beneficial effects on a variety of other functions in neutrophils from healthy volunteers.^10 11^ Evidence from case reports, small case series and small clinical trials suggested recombinant IFN-γ may improve outcomes in some critically ill patients with sepsis or invasive fungal disease.^12^,^15^ In contrast, a recent clinical trial found that IFN-γ did not reduce the incidence of hospital-acquired pneumonia (HAP) and instead was associated with an increased rate of HAP.^16^

The primary aim of this study was to determine the effect of ex vivo application of IFN-γ on neutrophils from critically ill patients. In previous studies, dysfunctional neutrophil phagocytosis, defined as <50% of isolated neutrophils being capable of ingesting 2 or more zymosan particles, has been independently associated with significantly increased risk of ICU-AI in critically ill patients.^2^ A secondary aim was to gain insights into mechanisms by which IFN-γ may improve function in patients’ neutrophils. FcγRI, the high affinity IgG receptor and key phagocytic receptor, has been shown to be upregulated transcriptionally and at the cell surface of neutrophils following in vivo treatment with IFN-γ.^17 18^ FcγRI and other key FcγRs (IIa and IIIb) signal through a variety of pathways; however, the involvement of phosphoinositide 3-kinase (PI3K) is key to many effector functions following FcγR engagement.^19 20^ We, therefore, focused attention on FcγR engagement and PI3K activity.

## Materials and methods

### Materials

Dextran was from Pharmacosmos (Holbaek, Denmark), Percoll Plus from Cytiva (Little Chalmont, UK) and Iscove’s Modified Dulbecco’s Medium from Life Technologies (Paisley, UK). AS605420 (a phosphoinositide 3-kinase gama (PI3K-γ) isoform-specific inhibitor with a reported IC_50_ of 8 nM^21^) was from Tocris (Abingdon, UK). Hanks’ balanced salt solution, Triton X-100, zymosan A from *Saccharomyces cerevisiae*, Tris HCl, NaCl, EDTA, Nonidet NP-40, phenylmethylsulphonyl fluoride, sodium fluoride (NaF), sodium orthovanadate (Na_3_VO_4_), protease inhibitor cocktail, dihydrorhodamine 123 (DHR123) and Giemsa staining solution were from Sigma Aldrich Ltd (Gillingham, UK). TruStain FcX, murine fluorescein isothiocyanate (FITC)-conjugated anti-human CD11b (clones ICRF44 and CBRM1/5 for the activated form), PE-conjugated anti-human CD64 (clone 10.1), FITC-conjugated anti-human CD32 (clone FUN-2) and Alexa fluor 647-conjugated anti-human CD16 (clone 3G8) monoclonal antibodies, and their respective murine isotype controls, were from Biolegend (London, UK). Cdc42 and Rac1,2,3 G-LISA Activation Assays (ELISA kits measuring GTPase activity that binds specifically to the GTP-bound form of the relevant proteins) were from Cytoskeleton Inc (Colorado, USA). Total Rac and Cdc42 (human) ELISA kits were from antibodies.com (Cambridge, UK). A clinical strain of *Staphylococcus aureus* was a kind gift from Professor John Perry (Microbiology Department, Freeman Hospital, Newcastle upon Tyne). Recombinant human IFN-γ was purchased from R&D Systems (Abingdon, UK).

### Patients

Patients were recruited from three general ICUs (Freeman Hospital, Newcastle upon Tyne, Royal Victoria Infirmary, Newcastle upon Tyne and Sunderland Royal Hospital) and one neurosurgical ICU (Royal Victoria Infirmary, Newcastle upon Tyne). Inclusion criteria were admission to the ICU with the clinical expectation of remaining there and surviving for over 24 hours, provision of written informed consent (either from the participant or from a personal or professional consultee if the patient lacked capacity) and the requirement for organ support, comprising one or more from intubation and mechanical ventilation, non-invasive respiratory support (non-invasive ventilation or high-flow nasal cannula oxygen), intravenous vasopressor support or haemofiltration).

Exclusion criteria comprised age under 18 years, pregnancy, known infection with HIV, haematological malignancy or chronic use of immunosuppression (other than corticosteroids at a maximum dose of 10 mg prednisolone daily or equivalent). Acute prescription of corticosteroids for acute illness at daily doses equivalent to up to 200 mg hydrocortisone was permitted.

### Isolation of neutrophils from peripheral blood

In all experiments described, whole blood was collected into VACUETTE TUBE 9 mL 9NC Coagulation tubes, containing sodium citrate 3.2%. Neutrophils were isolated from whole blood by dextran sedimentation and fractionation through discontinuous Percoll gradients as previously described.^22^ Neutrophils were only used in experiments if viability was >99% (assessed by trypan blue exclusion) and purity >95% (assessed by morphological analysis).

### Phagocytosis of zymosan by isolated neutrophils

Zymosan particles were opsonised for 30 min at 37°C using autologous serum as described previously.^23^ Phagocytosis was quantified as the proportion of neutrophils containing two or more zymosan particles as assessed by light microscopy. Values of less than 50% were defined as dysfunctional. Neutrophil dysfunction is associated with an increased risk of ICU-AI.^2^ Isolated neutrophils were incubated with AS605420 (10 nM) or TruStain FcX for 30 min at 37°C before incubation with IFN-γ (58.3 pM, 1 ng/mL) for 1 hour at 37°C prior to the addition of zymosan particles.

### Killing of bacteria by isolated neutrophils

The clinical isolate used was a methicillin-sensitive *S. aureus* derived from a respiratory sample taken from a patient with suspected ventilator-associated pneumonia (VAP) (strain 97STA). The loss of viability of *S. aureus* (opsonised in autologous serum) over time was measured using a previously described method.^24^ Briefly, following 30 min of incubation with AS605420 (10 nM) or TruStain FcX, neutrophils were exposed to IFN-γ (1 ng/mL) for a further hour at 37°C. Bacteria were opsonised with serum from the patients donating neutrophils. The neutrophils were mixed with live, serum-opsonised bacteria for 30 min at 37°C, then lysed with Triton X100. Serial dilutions were performed before plating 100 µL on duplicate LB agar plates. After an overnight incubation at 37°C, the number of colonies was assessed and expressed as colony forming units/mL. Results were expressed as the percentage of bacterial growth following the 30 min incubation, relative to a control where bacteria were incubated alone.

### Intracellular reactive oxygen species release by neutrophils in whole blood

Reactive oxygen species (ROS) production was assessed by flow cytometry. Briefly, 300 µL of whole blood from each patient was diluted with phosphate-buffered saline (PBS) without calcium or magnesium (1:5 dilution) and incubated with DHR 123 (1 µM) at 37°C for 15 min. Following this, diluted, stained whole blood was incubated with AS605420 (10 nM) for 30 min at 37°C before 1 hour of exposure to IFN-γ (1 ng/mL). The sample was then stimulated with phorbol myristate acetate (1 µM) for 15 min at 37°C. After red cell lysis with BD FACS Lysing Solution 1x and washes with PBS containing 2% bovine serum albumin, each sample was acquired using a BD FACSymphony flow cytometer (Becton Dickinson Biosciences), gating on live neutrophils using forward and side scatter, with DAPI (4',6-diamidino-2-phenylindole) staining and analysed using FCS Express 7 (De Novo Software). Results are expressed as the change in median fluorescence intensity compared with unstimulated control.

### Neutrophil cell surface marker analysis via flow cytometry

Whole blood was used to stain for CD11b (ITGAM), CD16 (FcγRIII), CD32 (FcγRII) and CD64 (FcγRI). 100 µL of whole blood was incubated at 37°C for 1 hour with IFN-γ (1 ng/mL) in a shaking incubator prior to incubation with each antibody for 30 min at room temperature. Samples were then acquired following the procedure described in the previous subsection.

### Rho GTPase activity

Freshly isolated adherent neutrophils were incubated with IFN-γ (1 ng/mL), AS605420 (10 nM), TruStain FcX or medium alone. For each condition, either no zymosan particles (control) or zymosan particles were added for 5 min prior to preparation of cell lysates, as previously described.^25^ Rac 1,2,3 and Cdc42 activity were assessed by Rac 1,2,3 and Cdc42 G-LISA Activation Assay Kits respectively. Total Rac and Cdc42 were evaluated using ELISA kits.

### Statistical analysis

The primary outcome for the study was the difference in phagocytosis in dysfunctional neutrophils in the presence of IFN-γ or control. We assumed a median phagocytosis of 30% (SD 10%) in the control group and 50% in the treatment group (SD 10%). With 90% power and alpha 5%, a paired sample size of 5 was required. To allow for heterogeneity in ICU populations and potential dropouts, we recruited sample sizes of at least 6 for all experiments.

All analyses were performed using GraphPad Prism V.10 (GraphPad, La Jolla, California, USA). The statistical tests used are described in the figure legends. A p<0.05 was considered statistically significant.

## Results

Characteristics of the total of 31 patients studied are shown in table 1. Online supplemental table describes the reasons for ICU admission in the 31 patients. In the results that follow, neutrophil dysfunction refers to<50% of neutrophils ingesting ≥2 zymosan particles in a zymosan phagocytosis assay (n=23).^2^ The figures and text below describe differing numbers of patients as not every patient was studied in each experiment.

**Table 1:** Demographic and clinical data for critically ill patients with functional and dysfunctional neutrophils

	Functional (n=8)	Dysfunctional (n=23)
Median age, years (range)	61 (40–77)	61 (20–77)
Male gender (%)	4 (50)	12 (52)
Ethnicity (% white)	100	100
Median APACHE II score (range)	23 (5–29)	25 (10–40)
Sepsis (%)	4 (50)	13 (57)
Median clinical frailty score (range)	2 (2–4)*	3 (2–6)†
Survival to ICU discharge (%)	6 (75)	15 (65)
Clinical status (%)		
Intubated and ventilated	7 (88)	20 (87)
Inotrope support	3 (38)	12 (52)
Haemofiltration/dialysis	1 (13)	3 (13)
Smoking status (%)		
Current/Ex	3 (38)	9 (43)‡
Never	5 (63)	12 (57)
Reason for ICU admission§		
Medical	3 (38)	11 (48)
Surgical	3 (38)	12 (52)
Trauma	2 (25)	0
Presence of specific comorbidities (%)		
Chronic kidney disease 3+	2 (25)	1 (4)
Type 2 diabetes	0	7 (30)
Hypertension	3 (38)	8 (35)
Airways disease (COPD, asthma)	2 (25)	4 (17)
Current or previous alcohol excess	2 (25)	3 (13)
Ischaemic or valvular heart disease	1 (13)	2 (9)
Key medications		
Acute immunosuppressants¶	1 (13)	5 (22)
Anti-infectives	6 (75)	19 (83)

* Data missing from one participant.

† Data missing from two participants.

‡ Data missing from two participants.

§ Individual reasons for ICU admission are shown in the separate online supplemental table.

¶ Acute immunosuppressant prescriptions included prednisolone 40 mg once daily, dexamethasone 6.6 mg two times per day and hydrocortisone up to a maximum daily dose of 200 mg.

APACHE II, Acute Physiology and Chronic Health Evaluation; COPD, chronic obstructive pulmonary disease; ICU, intensive care unit.

### Impairment of neutrophil phagocytosis by critical illness is restored by IFN-γ treatment

**Figure 1:**
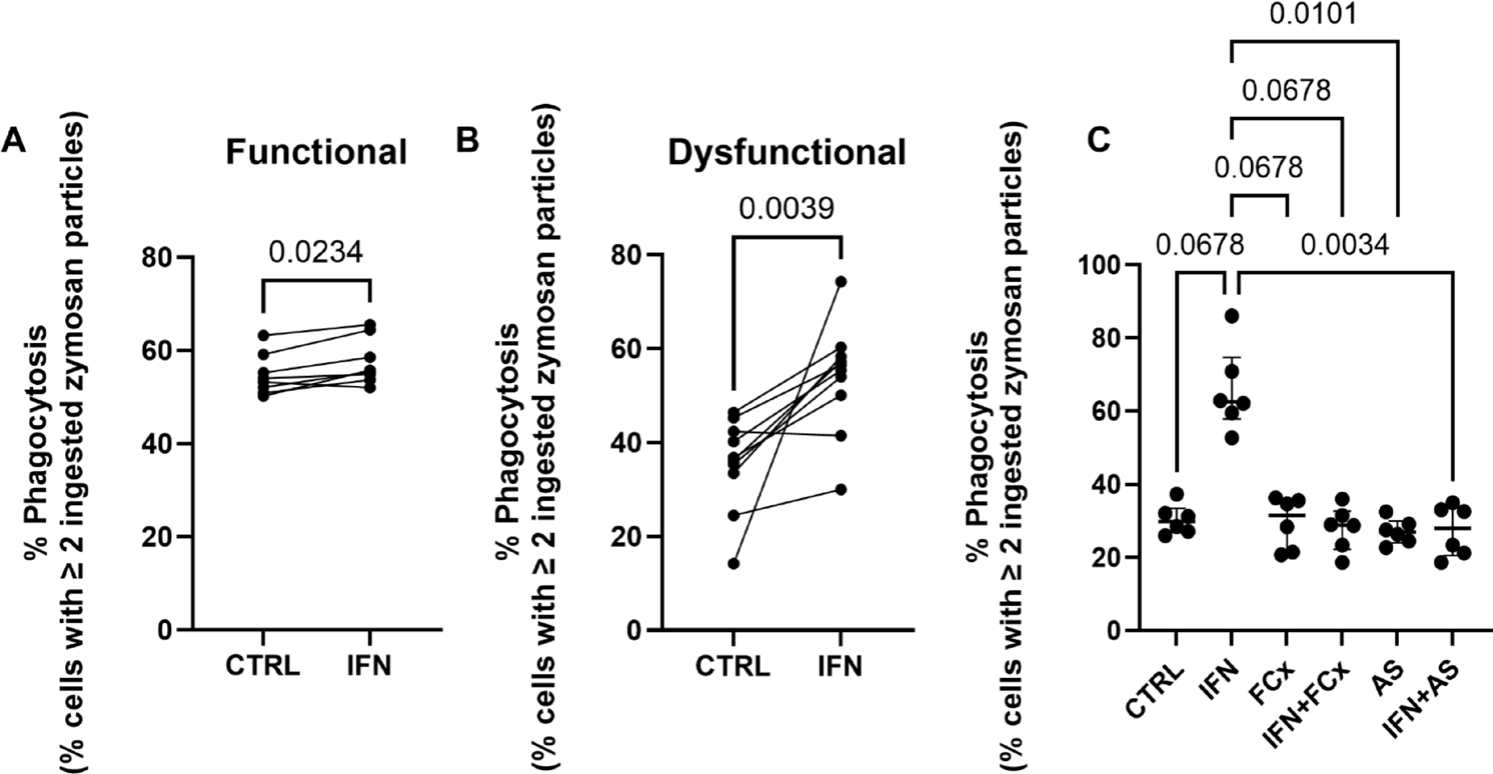
Neutrophil phagocytosis in critically ill patients is improved by IFN-γ treatment. (**A, B**) Neutrophil phagocytosis was measured and patients characterised on the basis of whether neutrophils were functional or dysfunctional as described in the Methods section. Dysfunction is defined as <50% of neutrophils ingesting ≥2 zymosan particles. Neutrophils from patients were incubated with IFN-γ (1 ng/mL) or control for 1 hour and phagocytosis of zymosan assessed. (**C**) Neutrophils from patients with dysfunctional neutrophils were preincubated with a PI3K-γ inhibitor AS (AS 605420, 10 nM) or FcX (Fc block) for 30 min before the application of IFN-γ (1 ng/mL) for 1 hour. Bars represent medians. (**A**) Wilcoxon signed rank test (n=8). (**B**) Wilcoxon t-test (n=1). (**C**) Friedman test with Dunn’s multiple comparisons test (n=6). Comparisons shown are against the second (IFN-γ) column.

Incubation of functional neutrophils (ie, ≥50% of neutrophils ingesting ≥2 zymosan particles) with IFN-γ resulted in a small augmentation of phagocytosis of autologous serum-opsonised zymosan particles (figure 1A). However, phagocytic augmentation by IFN-γ was more pronounced in dysfunctional neutrophils from patients (figure 1B). Due to this, all further mechanistic work focused on patients with dysfunctional neutrophils.

### PI3K-γ and FcγRs are involved in reversal of dysfunctional neutrophil phagocytosis in critical illness by IFN-γ

Following IFN-γ exposure, several signalling pathways are activated. IFN-γ increases the expression of neutrophil CD64 (FcγRI) in vivo.^26^ FcγR engagement activates PI3Ks in neutrophils.^27^ Therefore, inhibitors of both PI3K-γ and FcγRs were applied ex vivo to understand the mechanism behind IFN-γ-mediated improvement of neutrophil function. Inhibiting PI3K-γ with an isoform-specific inhibitor or blocking FcγRs with TruStain FcX (figure 1C) prevented IFN-γ from restoring phagocytosis.

### IFN-γ treatment improves bacterial killing and ROS production in dysfunctional neutrophils from critically ill patients

**Figure 2:**
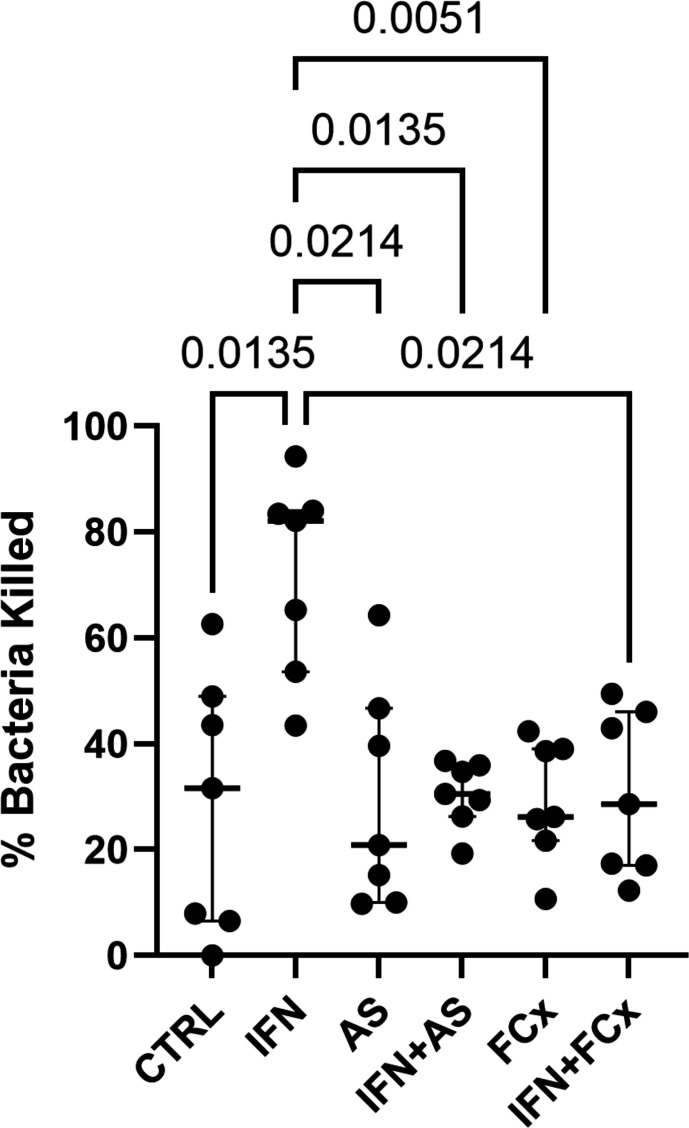
IFN-γ improves bacterial killing in dysfunctional neutrophils from critically ill patients. Percentage of *Staphylococcus aureus* killed by neutrophils isolated from seven critically ill patients with neutrophil dysfunction. Dysfunctional neutrophils were preincubated with Fc block (FcX) or a PI3K-γ inhibitor (AS605420 or ‘AS’, 10 nM) for 30 min before the application of IFN-γ (1 ng/mL) for 1 hour. Friedman test with Dunn’s multiple comparisons test. Bars represent medians. Comparisons shown are against the second (IFN-γ) column.

**Figure 3:**
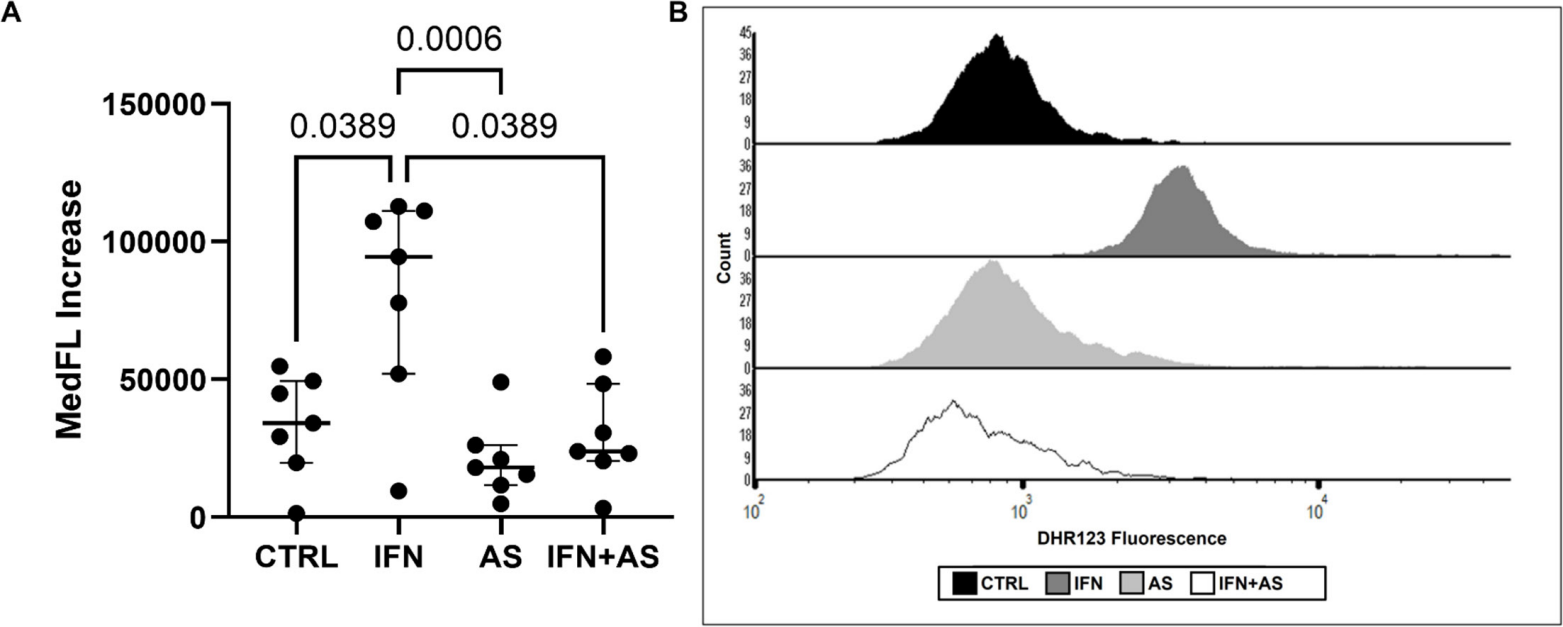
IFN-γ increases intracellular ROS release in dysfunctional neutrophils from critically ill patients. Whole blood was incubated with 1 µM DHR123 before incubation with 10 nM AS605420 for 30 min prior to addition of 1 ng/mL IFN-γ for 1 hour and subsequent stimulation with 1 µM PMA. (**A**) Data are expressed as fluorescence intensity and shown as individual points from 7 patients. Bars represent medians. Friedman test with Dunn’s multiple comparisons test. (**B**) Representative histogram of DHR fluorescence generated using FCS Express. DHR, dihydrorhodamine; MedFL, median fluorescence intensity; PMA, phorbol myristate acetate; ROS, reactive oxygen species.

IFN-γ enhanced killing of *S. aureus* by dysfunctional neutrophils from critically ill patients (figure 2). This effect was abrogated by FcγR blockade and by PI3K-γ inhibition (figure 2). ROS generation was also more pronounced following IFN-γ treatment (figure 3A,B). Inhibiting PI3K-γ profoundly impacted the IFN-γ-mediated improvement in intracellular ROS release (figure 3A,B).

### Surface CD64 expression increases on dysfunctional neutrophils following IFN-γ treatment

**Figure 4:**
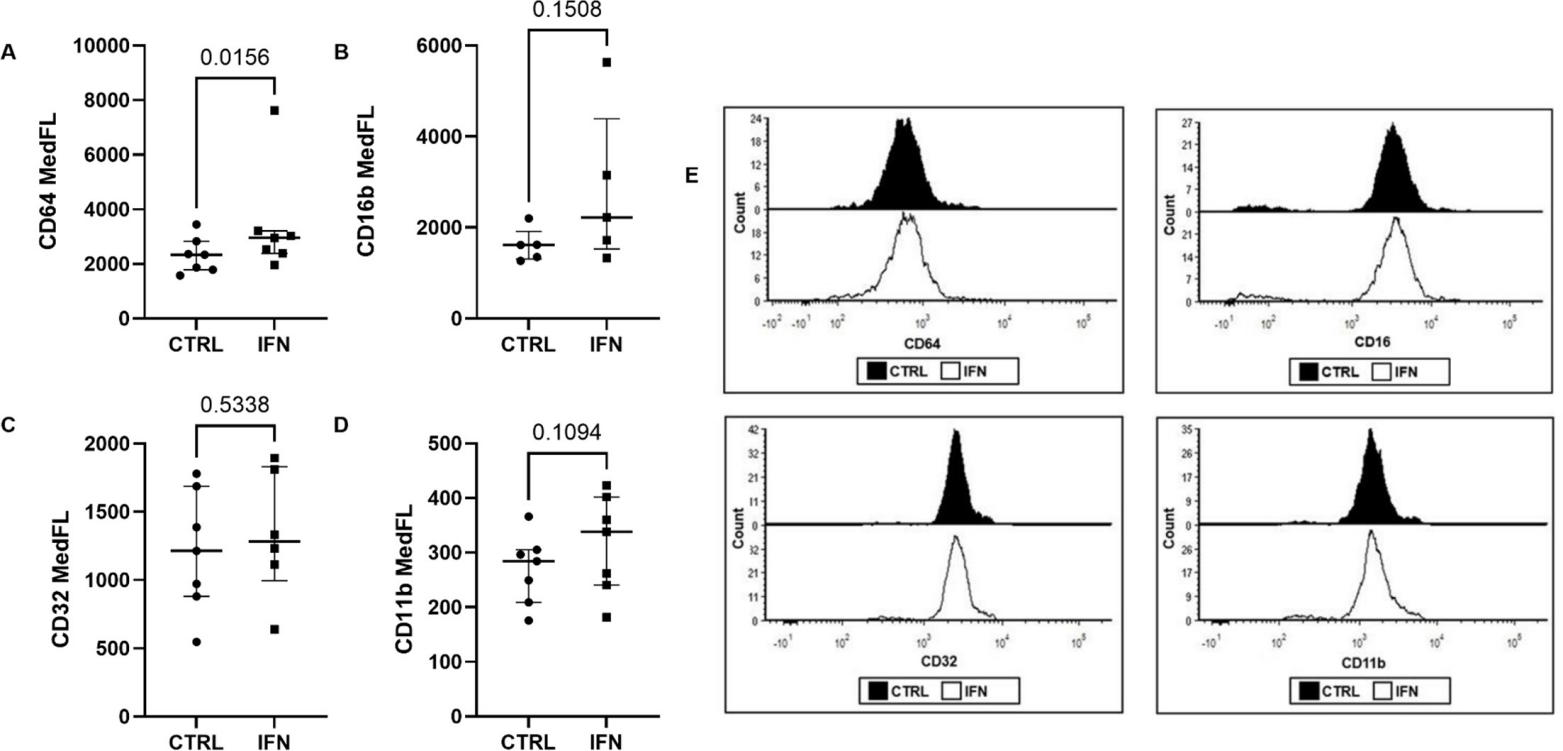
Cell surface expression of phagocytic receptors on dysfunctional neutrophils after incubation of whole blood with IFN-γ. Expression of neutrophil CD64 (**A**), CD16 (**B**), CD32 (**C**) and CD11b (**D**) was measured by flow cytometry following incubation of whole blood with IFN-γ (1 ng/mL) for 1 hour. Wilcoxon signed rank test, n=7. (**E**) Representative fluorescence histogram for each marker, generated using FCS Express. MedFL, median fluorescence intensity.

Flow cytometric analysis of neutrophil surface markers in whole blood revealed little change in expression of complement receptor 3 (CR3, CD11b) and FcγRs after incubation with IFN-γ (figure 4A,B). A small, statistically significant increase in CD64 (FcγRI) expression was observed following incubation of whole blood with IFN-γ for 1 hour (figure 4A).

### IFN-γ-mediated improvement of neutrophil phagocytosis is associated with an increase in small GTPase activity

**Figure 5:**
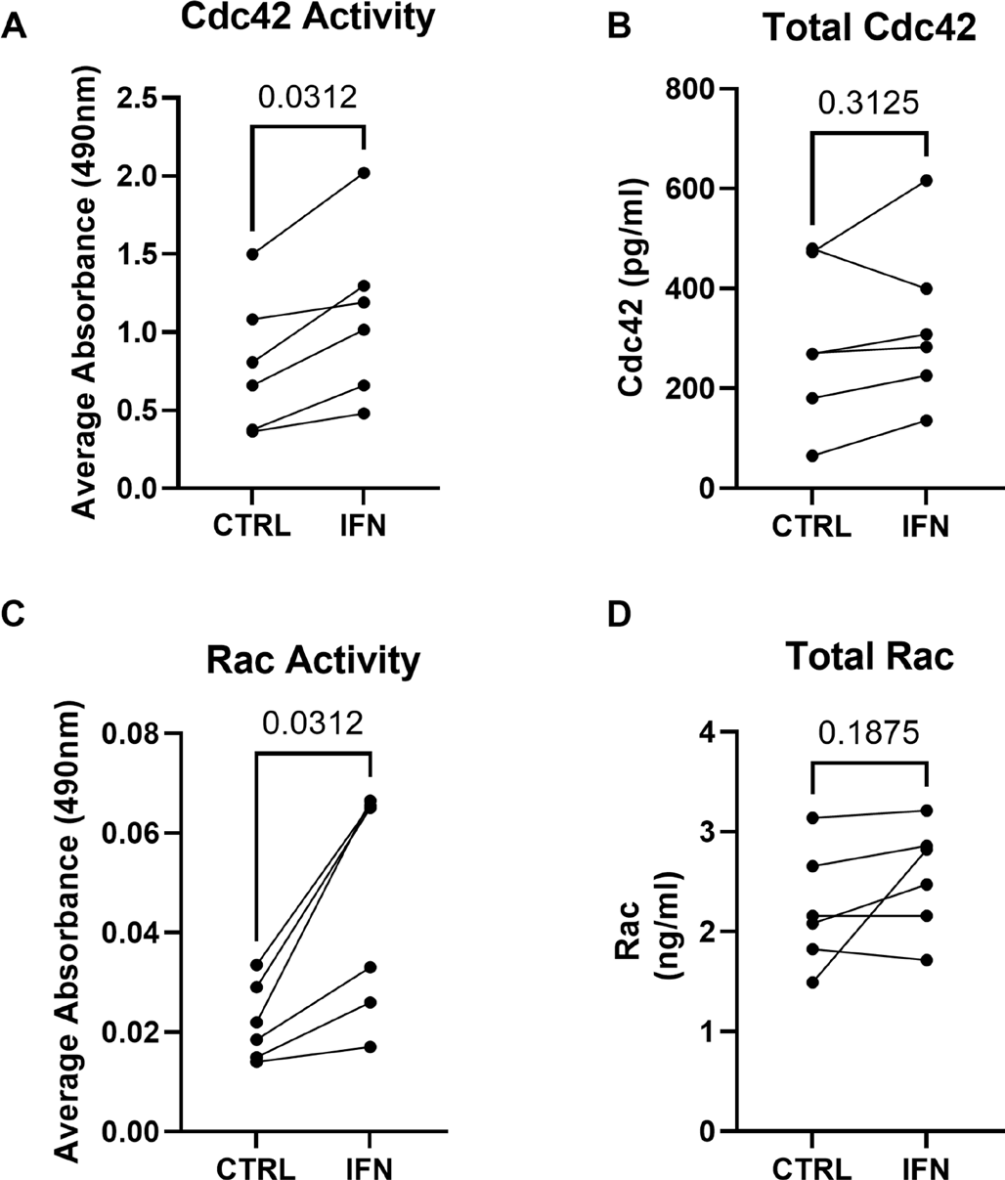
IFN-γ increases Rac and Cdc42 activity in dysfunctional neutrophils from critically ill patients. IFN-γ (1 ng/mL for 1 hour) or vehicle was added to dysfunctional neutrophils from critically ill patients (n=6). (**A**) Cdc42 and (**C**) Rac 1,2,3 activity was then assessed by G-LISA. Results are expressed as absorbance values at 490 nm. (**B**) Total Cdc42 and (**D**) total Rac were assessed by ELISA. Wilcoxon signed rank test.

Given the involvement of the small GTPases Rac and Cdc42 in FcγR-mediated actin assembly and phagocytosis,^28^ their activity was assessed by G-LISA. IFN-γ-treated neutrophils showed a significant increase in activity of both Cdc42 (figure 5A) and Rac (figure 5C), respectively. Total Cdc42 (figure 5B) and Rac (figure 5D) were also measured in neutrophils with and without ex vivo exposure to IFN-γ. There was no significant increase in the total expression of either Rac or Cdc42 in neutrophils following IFN-γ treatment.

## Discussion

The data presented suggest that IFN-γ applied ex vivo can significantly restore function to neutrophils from critically ill patients. The effect was more pronounced in neutrophils that were already dysfunctional, which identifies patients at highest risk of ICU-AI. In neutrophils from these patients, IFN-γ improved the key functions of phagocytosis, bacterial killing and intracellular ROS release. Nevertheless, a small, statistically significant improvement in phagocytosis was also observed when IFN-γ was applied to patients’ neutrophils that retained adequate function. This is consistent with the findings of others showing receptor-mediated phagocytosis and microbial oxidative killing ability are increased by IFN-γ in neutrophils from healthy volunteers.^10 11^

Our data suggest that IFN-γ-mediated improvement of bacterial killing in critically ill patients’ dysfunctional neutrophils is dependent on FcγRs. This important role for FcγRs in IFN-γ-mediated improvement of neutrophil pathogen clearance seems unlikely to depend on de novo transcriptional changes, given the effect was observed within an hour. However, transcriptomic data from a myeloid cell line showed that IFN-γ can stimulate expression of genes modulating phagocytosis, bacterial killing, chemotaxis and other classical neutrophil functions.^29^ FcγR-mediated pathogen clearance did not appear to be attributable to increased FcγR expression at the cell surface, as IFN-γ only induced small increases in CD64 in flow cytometric analysis. Others have described that exposure to IFN-γ in healthy volunteers led to a rise in expression in neutrophil CD64.^26^

Our data also suggest that PI3K-γ is required for IFN-γ-mediated phagocytosis, killing of *S. aureus* and ROS generation in neutrophils from critically ill patients. This implies complex regulation of PI3Ks in this setting, as previous work has implicated the delta isoform of PI3K in driving impaired phagocytosis and bacterial killing in neutrophils from critically ill patients.^25^

The restoration of phagocytosis mediated by FcγRs and/or PI3K-γ appeared to be associated with increased activity of the small Rho GTPases Rac and Cdc42. The complex interactions between PI3K signalling and Rho GTPases have been reviewed in detail by McCormick *et al*,^30^ and this field deserves further attention. In macrophages, PI3Ks appear to regulate cycling of the Cdc42 activity required for FcγR-mediated phagocytosis.^31^

Elegant human challenge models have suggested IFN-γ can reverse immunosuppression induced by repeated lipopolysaccharide challenge.^32^ Increased clinical experience of using IFN-γ for licensed clinical indications such as CGD and malignant osteopetrosis^7 8 33^ has encouraged greater exploration of IFN-γ as a rescue therapy in severe sepsis or invasive fungal disease. A small landmark trial by Döcke *et al* demonstrated upregulation of monocyte HLA-DR in parallel with clinical improvement in patients with sepsis receiving IFN-γ.^12^ Suppressed monocyte HLA-DR has been widely used as a biomarker of immune suppression in sepsis and critical illness,^34^ and the reproducible capacity of IFN-γ to restore HLA-DR has further driven enthusiasm for its use in sepsis and as an adjunct in preventing or treating invasive fungal disease.^13^,^1535^

However, the cumulative data suggesting a potential role for IFN-γ as a therapeutic in critical care must be balanced against the heterogeneity of critically ill patients and the complexity of IFN-γ’s role in the pathogenesis of critical illnesses. There is an increasing recognition that well-defined hyperinflammatory and hypoinflammatory endotypes stratify risk and potential treatment-responsiveness in acute respiratory distress syndrome^40^ and ‘omics-based approaches may usher in a precision medicine strategy for the management of sepsis endotypes.^41 42^ An IFN-γ-driven sepsis endotype has been associated with increased mortality.^43^ Furthermore, defined polymorphisms in *IFNG*, the gene encoding IFN-γ, can produce variations in circulating levels of IFN-γ^44^ and have been associated with increased risks of infection,^45 46^ though not, to our knowledge, in ICU-AI. While keeping in mind the heterogeneity in ICU populations and the complexity of IFN-γ production in individuals, our approach suggests that critically ill patients with demonstrable, acquired innate immune dysfunction may potentially benefit from IFN-γ, but this requires testing in randomised, blinded, placebo-controlled clinical trials.

The only sizeable, reported randomised control trial studying IFN-γ as a potential treatment in critically ill patients to date was stopped early due to a suggestion of harm associated with IFN-γ.^16^ The trial included a high proportion of patients with trauma and traumatic brain injury who are at a high risk of VAP.^47 48^ Further trials are currently exploring the potential of IFN-γ to reduce antibiotic use in patients with low mHLA-DR at particularly high risk of ICU-AI (ISRCTN 10449048).

While we believe the demonstrated improvement in three key neutrophil effector functions by IFN-γ to be potentially important, we are aware that there are limitations to this study. We did not quantify plasma IFN-γ. We only assessed the effect of IFN-γ on killing of a single, Gram-positive pathogen. *S. aureus* was selected for study because it is an important source of nosocomial infection. In UK ICUs, we found *S. aureus* to be the leading cause of VAP, the ICU-AI associated with highest mortality and cost.^49^ Also, pharmacological inhibitor studies are constrained by the absence of absolute specificity, and we cannot exclude off-target effects in our mechanistic studies. However, difficulties in genetically modifying human neutrophils leave pharmacological studies relatively attractive at present.

Furthermore, the reasons for admission to ICU in our cohort of patients were heterogeneous and our patient population was relatively small. Patients with dysfunctional neutrophils had lower ICU survival, less chronic kidney disease, more baseline diabetes and more use of immunosuppressants during their ICU stay (table 1), and we cannot completely exclude the possibility that results may have been influenced by these relatively small differences in baseline factors. In addition, the cohort was composed entirely of white participants and so did not reflect the general demographic of the UK.

In summary, IFN-γ reverses the impaired neutrophil function associated with increased risk of ICU-AI in critically ill patients and FcγRs and PI3K-γ are implicated in the functional improvement. Further work is required to determine if these beneficial effects can be safely translated into benefits for patients at highest risk of ICU-AI.

## Supplementary material

10.1136/thorax-2025-223280 online supplemental table 1

10.1136/thorax-2025-223280 online supplemental file 1

## Data Availability

Data are available on reasonable request.
