# Supplementary material for: Interferon gamma applied ex vivo restores function to neutrophils from critically ill patients
Source: Thorax. 2025 Aug 28;81(4):e223280. doi: 10.1136/thorax-2025-223280 (PMC13018806; doi:10.1136/thorax-2025-223280)
Supplement: online supplemental table 1 [file thorax-81-4-s001.pdf]

Participant number	Group	Cause of ICU admission	Classification
1	Functional	Rib fractures and respiratory failure	Trauma
2		Seizure	Medical
3		Respiratory failure	Medical
4		Guillain-Barré syndrome	Medical
5		Complications following Hartmann's procedure	Surgical
6		Head injuries	Trauma
7		Hepatobiliary surgical intervention	Surgical
8		Neurosurgical intervention post stroke	Surgical
9	Non functional	Respiratory arrest and type 2 respiratory failure	Medical
10		Complications post cervical decompression and fusion	Surgical
11		Suspected central nervous system infection	Medical
12		Bleeding duodenal ulcer and perforated bowel	Surgical
13		Inpatient cardiac arrest	Medical
14		Seizure	Medical
15		Monitoring following emergency laparotomy	Surgical
16		Cardiac arrest secondary to ST-elevation myocardial infarction	Medical
17		Severe metabolic derangement	Medical
18		Haematemesis	Medical
19		Complications following ureteric stone surgery	Surgical
20		Asthma exacerbation	Medical
21		Complications following elective aorta repair	Surgical
22		Complicated pancreatitis	Surgical
23		Complications following loop gastric bypass	Surgical
24		Complications following Hartmann's procedure and subtotal colectomy	Surgical
25		Complications following anterior resection	Surgical
26		Pneumonia	Medical

27	Complicated pancreatitis	Surgical
28	Diabetic ketoacidosis	Medical
29	Intestinal failure	Medical
30	Complications following laparoscopic cholecystectomy	Surgical
31	Retropharyngeal abscess	Surgical
