# Supplementary material for: Interferon gamma applied ex vivo restores function to neutrophils from critically ill patients
Source: Thorax. 2025 Aug 28;81(4):e223280. doi: 10.1136/thorax-2025-223280 (PMC13018806; doi:10.1136/thorax-2025-223280)
Supplement: online supplemental file 1 [file thorax-81-4-s002.pptx]

## Slide 1
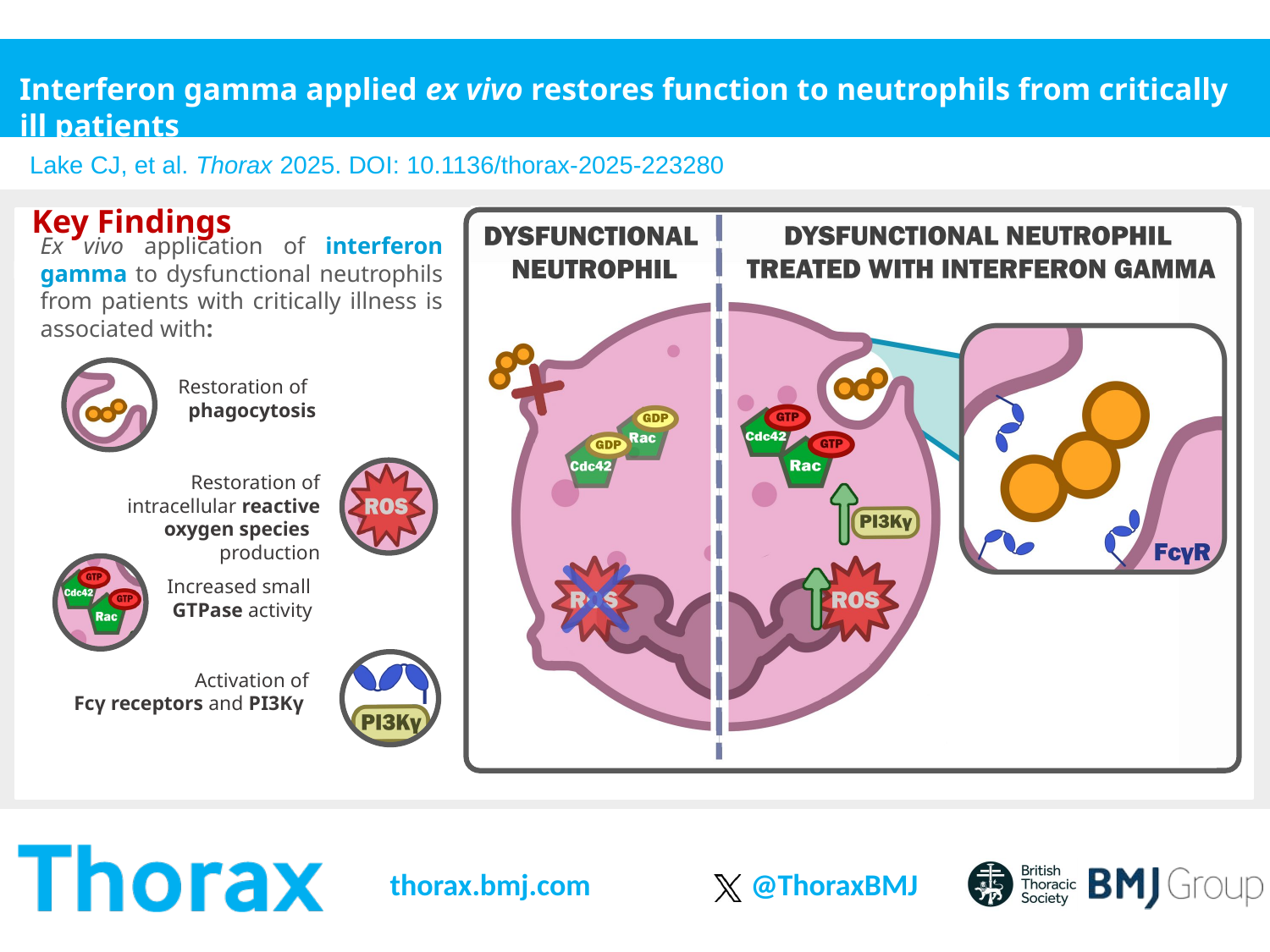

Interferon gamma applied ex vivo restores function to neutrophils from critically ill patients
Lake CJ, et al. Thorax 2025. DOI: 10.1136/thorax-2025-223280
Key Findings
Ex vivo application of interferon gamma to dysfunctional neutrophils from patients with critically illness is associated with:
Manuscript Title
Restoration of
 phagocytosis
Restoration of intracellular reactive oxygen species . production
Increased small
 GTPase activity
Activation of
Fcγ receptors and PI3Kγ.
© Author(s) (or their employer(s) 2019. Re-use permitted under CC BY. Published by BMJ.
thorax.bmj.com @ThoraxBMJ
